# Targeted Therapies Compared to Dacarbazine for Treatment of BRAF^V600E^ Metastatic Melanoma: A Cost-Effectiveness Analysis

**DOI:** 10.1155/2015/505302

**Published:** 2015-06-10

**Authors:** Vanessa Shih, Renske M. ten Ham, Christine T. Bui, Dan N. Tran, Jie Ting, Leslie Wilson

**Affiliations:** Department of Clinical Pharmacy, University of California, San Francisco, 3333 California Street, Laurel Heights, San Francisco, CA 94143, USA

## Abstract

*Purpose*. Two BRAF^V600E^ targeted therapies, dabrafenib and vemurafenib, have received US approval for treatment of metastatic melanoma in BRAF^V600E^ patients, a mutation that affects ~50% of patients. We evaluated the cost-effectiveness of BRAF inhibitors and traditional chemotherapy for treatment of metastatic melanoma. *Methods*. A Markov model was developed using a societal perspective. Transition probabilities were derived from two Phase III registration trials comparing each BRAF inhibitor against dacarbazine. Costs were obtained from literature, national databases, and Medicare fee schedules. Utilities were obtained from published literature. Deterministic and probabilistic sensitivity analyses were run to test the impact of uncertainties. *Results*. The incremental cost-effectiveness ratio of dabrafenib was $149,035/QALY compared to dacarbazine. Vemurafenib was dominated by dabrafenib. Probabilistic sensitivity analysis showed that, at a willingness-to-pay (WTP) threshold of ≤$100,000/QALY, dacarbazine was the optimal treatment in ~85% of simulations. At a WTP threshold of ≥$150,000/QALY, dabrafenib was the optimal treatment. *Conclusion*. Compared with dacarbazine, dabrafenib and vemurafenib were not cost-effective at a willingness-to-pay threshold of $100,000/QALY. Dabrafenib is more efficient compared to vemurafenib. With few treatment options, dabrafenib is an option for qualifying patients if the overall cost of dabrafenib is reduced to $30,000–$31,000 or a WTP threshold of ≥$150,000/QALY is considered. More comparative data is needed.

## 1. Introduction

Malignant melanoma continues to increase dramatically in incidence worldwide [1]. In 2013, an estimated 76,690 people in the US were diagnosed with melanoma of the skin, of whom about 9,480 will die of the disease [2]. The prognosis of patients with metastatic melanoma is very poor with the one-year survival rate ranging from 33 to 62%, depending on disease stage [3]. In patients diagnosed with melanoma, about 40–60% carry mutations in BRAF [4], which can lead to increased cellular proliferation and increased oncogenic cell activity [5]. Among these patients, 80–90% have the amino acid valine replaced with glutamic acid at codon 600 (BRAF^V600E^) [4, 6].

Until 2011, dacarbazine, a chemotherapeutic alkylating agent, was the standard of care for patients with metastatic melanoma despite its lack of survival benefit [7, 8]. However, due to improved knowledge on the molecular biology of melanoma and the discovery of oncogenic mutations contributing to disease progression, two novel therapies were developed [9]. In 2011, the US Food and Drug Administration (FDA) approved the BRAF kinase inhibitor vemurafenib (Zelboraf, Genentech, South San Francisco, CA) for treatment of metastatic melanoma in patients with the BRAF^V600E^ mutation. A second BRAF inhibitor, dabrafenib (Tafinlar, GlaxoSmithKline, Brentford, UK), was approved in 2013 for the same indication. Compared with palliative chemotherapy, BRAF inhibition demonstrated overall survival improvement and produced a response rate of nearly 50% in clinical trials [9, 10]. Additionally, BRAF inhibitors are oral agents and provide an improved side effect profile compared to chemotherapy.

However, BRAF inhibitor responses are not durable with development of resistance commonly occurring [11]. Furthermore, BRAF inhibitors come with a high cost burden so the question remains if these novel therapies provide value. The purpose of this study was to perform a cost-effectiveness analysis of the newly available oral targeted therapies vemurafenib and dabrafenib compared to each other and to dacarbazine in patients with unresectable stage III or stage IV metastatic melanoma positive for the BRAF^V600E^ mutation. To our knowledge, this is the first study to assess the comparative economic value of these therapies and results from this analysis may be used to inform decision making processes.

## 2. Methods

### 2.1. Study Population

Our study population was patients from two published Phase III clinical trials with unresectable stage III or stage IV metastatic melanoma positive for the BRAF^V600E^ mutation [4, 6]. Both were multicenter, randomized trials enrolling patients from twelve countries across Europe, North America, and Australia. The vemurafenib trial assigned 675 patients to either vemurafenib (*n* = 337, median age 56 years) or dacarbazine (*n* = 338, median age 52 years). We used the trial overall survival data results that were used by the FDA for drug approval and were available at the time of our study, begun in October 2013. Median follow-up time for the vemurafenib group was 3.8 months and the dacarbazine group 2.3 months. The dabrafenib trial assigned 250 patients to receive dabrafenib (*n* = 187, median age 53 years) or dacarbazine (*n* = 63, median age 50 years) and had a median time on study of 4.9 months. In both trials, patient populations had similar patient characteristics, disease severity, and disease progression. We did not use the results of the extended follow-up analysis published later because the longer follow-up time was longer for vemurafenib (12.5 months) and made the dabrafenib study noncomparable [21].

### 2.2. Model Overview

A Markov model was constructed to compare the cost-effectiveness of dabrafenib, vemurafenib, and dacarbazine using TreeAge Pro 2013 (TreeAge Software, Williamstown, MA, USA) for treatment of patients with stage IV or unresectable stage III metastatic melanoma with the BRAF^V600E^ mutation. This model was from a US societal perspective over the patient's remaining lifetime. We used 2013 USD and a societal willingness-to-pay (WTP) threshold of $100,000 per quality-adjusted life year (QALY).

**Figure 1:**
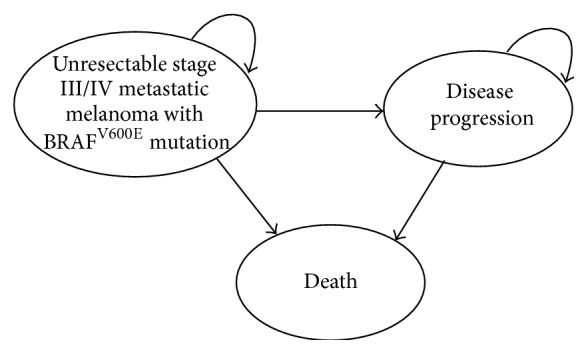
Markov structure diagramming progression of patients through health states.

Each monthly cycle of the model simulated the disease progression of a patient cohort through three discrete health states: stable disease, progression, and death (Figure 1). All patients started in stable disease and could remain with stable disease, move to progression, or die. Patients in the progression state could remain in progression or die (absorbing state).

Model outcomes were total treatment costs measured in 2013 US dollars and effect measured in QALY. Cost-effectiveness of each treatment was compared from least to most expensive using the incremental cost-effectiveness ratio (ICER) as defined by (Cost_treatment1_ − Cost_treatment2_)/(QALY_treatment1_ − QALY_treatment2_).

### 2.3. Model Inputs

**Figure 2:**
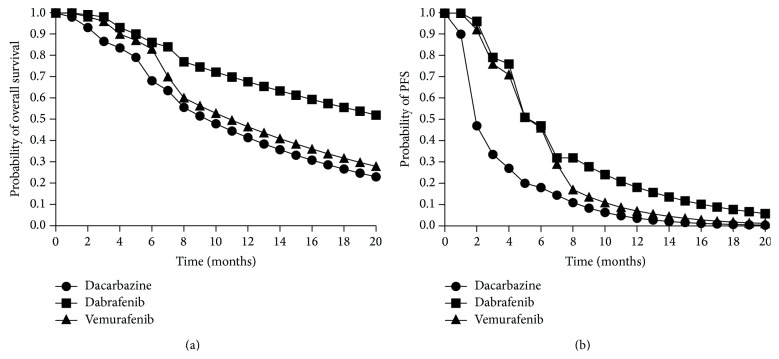
(a) Kaplan-Meier Overall Survival Curve. (b) Kaplan-Meier Progression-Free Survival Curve. (The probabilities from 0–8 months are from clinical trial data. The probabilities from 8 months to end of life are modeled using the DEALE method.)

Model inputs were clinical inputs including monthly health state transition probabilities, monthly side effect probabilities, and health state utilities to obtain QALYs, as well as cost inputs. Progression-free survival (PFS) for vemurafenib was derived from the published Phase III pivotal trial, while overall survival (OS) was obtained from the FDA summary basis for approval which was available at the time of this study [4, 22]. PFS and OS for dabrafenib were derived from the published Phase III pivotal trial [6]. Probability of death was 1-OS and probability of disease progression was OS-PFS. Dacarbazine was used as the comparator drug in both Phase III pivotal trials. Since patient characteristics of the dacarbazine treatment arms in both Phase III studies were similar, PFS and OS for dacarbazine were derived by averaging probabilities from both trials. The Declining Exponential Approximation of Life Expectancy (DEALE) method was used to extend the survival curves to represent a lifetime horizon (Figure 2) [23, 24]. DEALE is a good approximation when survival is short such as in these trials (10 months for vemurafenib and 9 months for dabrafenib). The hazard rate (HR) was calculated from the probability of overall survival and progression-free survival at month 8 in both trials and was then used to model mortality for the patient's remaining lifetime, assuming a constant HR.

Adverse event probabilities were obtained from published Phase III trials [4, 6]. All Grade III and Grade IV adverse events were included in the model along with Grade I and Grade II adverse events that were costly to treat or occurred with high frequency (Table 1). Adverse events included in the model fell into three categories: (i) hematological complications including neutropenia, thrombocytopenia, and leukopenia which occurred primarily in patients on dacarbazine; (ii) gastrointestinal adverse events including nausea, vomiting, and diarrhea which occurred across all treatment groups; (iii) skin adverse events including hyperkeratosis, skin papillomas, nonmelanoma skin cancers, and palmar plantar erythrodysesthesia (PPE) which affected primarily patients on dabrafenib and vemurafenib. Adverse events were assumed to need treatment independently of each other with the exception of nausea and vomiting which were assumed to occur together and require overlapping treatments.

Health utilities were included for quality adjustment expressed by QALYs. A drug-specific utility was calculated for both the stable disease and progression health states taking into account drug-specific rates of adverse events obtained from trial data (Table 1) [4, 6]. The proportion of the cohort experiencing each set of adverse events was multiplied by the utility weights for the adverse event states and summed to obtain the expected value of the utility for the health state specific to each drug. Utilities for melanoma health states were obtained from a published study that elicited utilities for metastatic melanoma specific health states from healthy volunteers in the United Kingdom and Australia [25]. Utilities for skin-related adverse event health states were taken from a catalog of dermatology utilities [26]. Utilities for non-skin-related adverse event states were obtained from other published studies [27]. A recent analysis demonstrating improved quality of life on dabrafenib compared with dacarbazine was not used as it did not provide real utilities required in a cost-effectiveness comparison [28].

Cost inputs included direct drug costs and costs of treating adverse events (Table 1). Drug costs were obtained from Redbook Online (Truven Health Analytics) and for dacarbazine this included costs for a prophylactic antiemetic regimen per National Comprehensive Cancer Network (NCCN) guidelines [12, 13]. In the dabrafenib trial, patients in the comparator arm could cross over to the treatment arm per protocol [6]. The vemurafenib trial protocol was later amended to allow for crossover as well [4]. To account for the crossover in the treatment costs, an additional cost equal to the cost of dabrafenib was included for all patients in the progression state in the dacarbazine treatment arm. Adverse event costs were obtained from literature [17, 29, 30], the Centers for Medicare and Medicaid Services (CMS) reimbursement fee schedules using Current Procedural Treatment (CPT) codes for physician procedural costs [15], and the Healthcare Cost and Utilization Project (HCUP) database for hospitalization costs [14].

### 2.4. Sensitivity Analysis

To evaluate the impact of drug cost on cost-effectiveness, a one-way sensitivity analysis was performed on the cost of dabrafenib and the cost of vemurafenib. Probabilistic sensitivity analysis on all variables was performed with a Monte Carlo simulation, randomly sampling 10,000 iterations, using confidence intervals where available, beta distributions for probabilities, and gamma distributions for costs. Using the Monte Carlo simulations, the cost-effectiveness acceptability curve was constructed plotting the percentage of iterations where each therapy was cost-effective against a range of WTP thresholds along the *x*-axis. The percentage of cost-effective iterations was calculated for each therapy by first calculating the net monetary benefit (NMB) at each WTP threshold (*λ*) using the equation NMB = *λ*  ×  (Total Effect − Total Cost) [31]. Then, for each treatment at each WTP threshold, the proportion of iterations where the treatment had the highest NMB provided the percentage of cost-effective iterations.

## 3. Results

### 3.1. Cost-Effectiveness Analysis

Total cost represents drug costs and toxicity management costs accrued over the patients' entire remaining lifetime. Drug costs were applied during all cycles in which patients were alive and side effect management costs, weighted by probability of occurring, were applied during each cycle in which patients received drug. Total cost was lowest for the dacarbazine treatment arm ($15,221), followed by dabrafenib ($38,547) and then vemurafenib ($49,937). Effectiveness of dacarbazine, vemurafenib, and dabrafenib was 0.1820, 0.2905, and 0.3385 QALYs, respectively (Table 2).

Dacarbazine was the least costly, but also least effective (Table 2). However, the ICER of dabrafenib, the next more costly option, relative to dacarbazine, was high, $149,035/QALY. This is not considered cost-effective, exceeding the current average US societal WTP of $100,000/QALY. Vemurafenib was dominated by dabrafenib because it was more costly than dabrafenib and less effective, demonstrating a strong preference for dabrafenib over vemurafenib for cost-effectiveness. In addition, the ICER of vemurafenib over dacarbazine chemotherapy was $319,972/QALY, clearly higher than any acceptable WTP threshold (Table 3).

### 3.2. Sensitivity Analysis

#### 3.2.1. Deterministic Sensitivity Analysis

One-way sensitivity analyses were performed on the cost of dabrafenib and vemurafenib in order to determine the effect of drug cost on cost-effectiveness. Using a WTP threshold of $100,000/QALY gained, dabrafenib only became a cost-effective option compared to dacarbazine when its cost was decreased to below $5,259/month, a ~30% decrease in monthly price. Vemurafenib was dominated by dabrafenib and was not cost-effective compared with dacarbazine.

#### 3.2.2. Probabilistic Sensitivity Analysis

**Figure 3:**
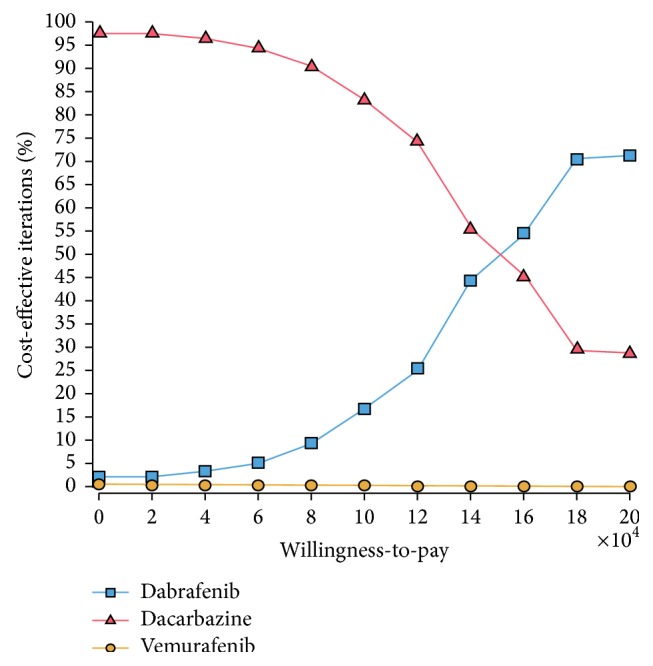
Cost-effectiveness acceptability curve. This acceptability curve shows, for each drug, which percentage of 10,000 Monte Carlo iterations is cost-effective over a range of WTP thresholds.

At a WTP threshold of $100,000/QALY, dacarbazine was the optimal treatment in about 85% of simulations while dabrafenib was the optimal treatment in about 15% of simulations (Figure 3). Thus, at this threshold, dacarbazine was the most cost-effective treatment despite its lower survival. At WTP threshold of ≥$150,000/QALY, dabrafenib became the optimal treatment, being cost-effective in the majority of iterations.

## 4. Discussion

We found that, at a societal WTP threshold of ≤$100,000/QALY, dacarbazine was the optimal treatment. At this threshold, neither dabrafenib nor vemurafenib was cost-effective relative to dacarbazine. The ICER high for dabrafenib and even higher for vemurafenib compared to dacarbazine was likely due to a large incremental cost for the two BRAF inhibitors with only a mild but important increase in QALY of about two months. The large incremental costs are primarily attributable to differences in drug costs; dabrafenib and vemurafenib are eight- and tenfold more expensive than dacarbazine even when including a prophylactic antiemetic regimen with dacarbazine. However, we also found that dabrafenib dominates vemurafenib, indicating strongly that dabrafenib is the more cost-effective choice between the two targeted therapies, which are becoming standard treatment.

A recently published study compared the cost-effectiveness of three treatment strategies, vemurafenib, ipilimumab, and dacarbazine [32]. They found an ICER for vemurafenib compared to dacarbazine of $353,993/QALY, which is comparable to our ICER for vemurafenib compared to dacarbazine of $319,972/QALY. Our study adds to the literature by comparing the two BRAF inhibitors and demonstrating superiority of dabrafenib over vemurafenib in terms of value.

Cost-effectiveness analysis (CEA) results may be used as indicators of appropriate drug pricing or as the basis of price negotiations between drug manufacturers and payers based on implicit thresholds which indicate a society's WTP for improvements in health given a fixed budget. In the US, commonly cited thresholds for coverage range from $50,000 to $100,000/QALY while benchmarking approaches suggest threshold values ranging from $20,000 to $358,000/QALY [33]. This threshold has not been obtained by appropriately ranking the ICERs of all treatments until a fixed budget is reached. Instead, ICERs are used to add new favorable interventions, under the thresholds, but without removing those above the thresholds. Therefore the best threshold is uncertain and should vary by each budget under consideration. If we use the WHO threshold of two to three times the per capita annual income, the US threshold would be between $110,000/QALY and $160,000/QALY [34]. Others who account for inflation in health care spending suggest a threshold of $200,000/QALY and $300,000/QALY [34]. It has been argued that this threshold should be increased for end-of-life treatments [35]. In the UK where coverage of new therapies is linked to a demonstration of cost-effectiveness under a £30,000/QALY threshold, new guidelines from NICE have allowed for approval of previously cost-inefficient therapies for treatments meeting the following criteria: for patients with a short life expectancy, extension of life by at least three months compared to the current NHS standard, and for a small patient population [36]. Approval is also appropriate if the appraisal committee believes the QALY weight that will drop the ICER below £30,000/QALY accurately represents public preferences [36].

From a US payer perspective, high drug costs create incentives to restrict use, but, from a provider perspective, these therapies provide life-extending opportunities and these benefits have been realized with one study demonstrating coverage of vemurafenib in at least 50% of plans examined when it was the only targeted therapy [37]. Nationally, this need has been recognized with the mandate from CMS that Medicare drug plans cover “all or substantially all” drugs in specific therapeutic classes, including anticancer agents [38]. Although our ICER for dabrafenib compared to dacarbazine is not cost-effective at that price under the traditional WTP threshold, as an end-of-life treatment for a disease state with few options, there is evidence to suggest that, at negotiated prices within specific health plans, it is within acceptable limits to US payers and can be recommended as a treatment option with value [35]. This acceptance reflects the recent editorial reviewing ICER thresholds which suggests using between $100,000/QALY and $150,000/QALY [34].

While novel targeted therapies may have a place in treatment algorithms, strategies to minimize their ICER and the subsequent budget impact on payers will increase the value of these products. Our sensitivity analyses also demonstrated that 30% price cuts are necessary to reduce the ICER of dabrafenib compared to dacarbazine to below the current WTP threshold holding all other factors constant. This highlights the need for price negotiations as well as patient assistance programs from the manufacturer to further increase these therapies' value proposition. Our study also demonstrated for the first time that if targeted therapies are chosen over chemotherapy, dabrafenib is clearly the cost-effective choice, dominating vemurafenib.

Our study has a few limitations. First, the sample size of the vemurafenib trial was three times larger than the sample size of the dabrafenib trial, potentially biasing the results in favor of dabrafenib by potentially presenting a lower incidence of adverse events. However, the large difference in drug cost between the two targeted therapies supports that the difference in adverse event probabilities and subsequently the difference in costs due to adverse events would not be the primary driver of differences in costs between dabrafenib and vemurafenib. Second, a recent study published the extended OS results of the Phase III vemurafenib trial which included the protocol amendment allowing crossover to a treatment arm as patients on dacarbazine progressed [21]. However, this was written into the protocol from the beginning for the dabrafenib trial and patients were analyzed per intent to treat. The crossover would likely lead to an overestimation of the effectiveness of dacarbazine and, in reality, we should see a larger incremental QALY for the targeted therapies. Considering this limitation, a larger incremental QALY may have resulted in a lower ICER. It would take an incremental effect of 0.2333 QALYs to bring the ICER to under the cost-effective threshold. Finally, our study did not include utilities for drug formulation which, if included, may lower the ICER of the oral targeted therapies. A previous study found that patients in the palliative care setting preferred oral chemotherapy to intravenous therapy [39] and this preference for oral treatment has been cited as a main driver for the increase in oral chemotherapy agents available [40]. Inclusion of these utilities would decrease the ICER for targeted therapies, potentially bringing them closer to the societal WTP threshold.

In conclusion, this analysis demonstrated that, at a strict WTP threshold of $100,000/QALY, dacarbazine is the most cost-effective treatment and that dabrafenib dominates vemurafenib. Since society has demonstrated a willingness to accept higher value thresholds in general and particularly for end of life treatments, we consider dabrafenib to have value in this disease state with very few options, especially after pricing negotiations specific to each healthcare system. These pioneer treatments come at a high cost for payers and determining the cost-effectiveness of these therapies will be paramount from a payer and provider perspective in order to make coverage as well as treatment decisions. This CEA is the first step in generating the evidence to support the clinically based NCCN recommendations of dabrafenib as first line treatment if a fixed budget and societal preference allow a WTP threshold of $150,000. Future studies should update this study with real-world comparative data on the effectiveness of these drugs as well as comparing the cost-effectiveness of combination treatment approaches which are now being considered.

## Figures and Tables

**Table 1:** Model inputs.

	Vemurafenib	Dabrafenib		Dacarbazine

*Base case adverse event probabilities by drug [4, 6] *				
Vomiting, nausea, Grades 1, 2	0.0023	0.0009		0.0106
Vomiting, nausea, Grades 3, 4	0.0010	0		0.0007
Diarrhea, Grades 1, 2	0.0230	0		0.0006
Diarrhea, Grades 3, 4	0.0005	0		0.0001
Hyperkeratosis, Grades 1, 2	0.0172	0.0113		0
Hyperkeratosis, Grades 3, 4	0.0010	0.0009		0
Skin papilloma, Grades 1, 2	0.0169	0		0
Skin papilloma, Grades 3, 4	0	0		0
Squamous cell carcinoma, Grades 1, 2	0	0.0018		0
Squamous cell carcinoma, Grades 3, 4	0.0105	0.0036		0
Keratoacanthoma, Grades 1, 2	0.0051	0		0
Keratoacanthoma, Grades 3, 4	0	0		0
Neutropenia, Grades 1, 2	0.0002	0		0.0019
Neutropenia, Grades 3, 4	0.0002	0.0004		0.0086
PPE, Grades 1, 2	0	0.0055		0
PPE, Grades 3, 4	0	0.0018		0
Leukopenia, Grades 1, 2	0	0		0.0013
Leukopenia, Grades 3, 4	0	0		0.0007
Thrombocytopenia, Grades 1, 2	0	0		0
Thrombocytopenia, Grades 3, 4	0	0		0.0020

	Base case value	Sensitivity analysis		Distribution
		Lower value	Upper value	

*Drug-specific utility values for each health state including side effects *				
Stable disease, dacarbazine	0.69	0	1	Beta
Progression, dacarbazine	0.45	0	1	Beta
Stable disease, dabrafenib	0.79	0	1	Beta
Progression, dabrafenib	0.52	0	1	Beta
Stable disease, vemurafenib	0.73	0	1	Beta
Progression, vemurafenib	0.49	0	1	Beta

*Monthly drug costs (2013 USD) *				
Dacarbazine cost per month, assuming one cycle per month and including administration and prophylactic antiemetic treatment costs [12–14]	$988.86	$678.29	$1356.85	Gamma
Dabrafenib monthly cost [6, 13]	$7569.60	$5677.20	$9462.00	Gamma
Vemurafenib monthly cost [4, 13]	$10807.40	$8105.55	$13509.25	Gamma

*Toxicity management costs per month on treatment *				
Hyperkeratosis, Grades 1, 2 [13, 15, 16]	$126.66	$114.26	$424.95	Gamma
Hyperkeratosis, Grades 3, 4 [13, 15, 16]	$1082.84	$1070.84	$1706.93	Gamma
Squamous cell carcinoma [15, 17]	$1595	$1128	$3408	Gamma
Nausea, vomiting BRAF inhibitor, Grades 1, 2 [12, 13]	$274.58	$274.58	$419.54	Gamma
Nausea, vomiting BRAF inhibitor, Grades 3, 4 [12–14]	$6855.76	$4480.05	$8009.39	Gamma
Diarrhea, Grades 1, 2 [13, 18]	$5.81	$5.81	$45.89	Gamma
Diarrhea, Grades 3, 4 [13–15, 18]	$7404.11	$3550.28	$7845	Gamma
Keratoacanthoma, Grades 1, 2 [6, 13, 15]	$113.67	$66.72	$181.31	Gamma
Skin papilloma, Grades 2, 3 [15]	$73	$61	$97	Gamma
PPE [13, 15, 19, 20]	$113.67	$113.67	$178.62	Gamma
Nausea, vomiting, dacarbazine, Grades 1, 2 [12, 13]	$84.66	$2.78	$4485.98	Gamma
Nausea, vomiting, dacarbazine, Grades 3, 4 [12–14]	$6665.84	$4208.25	$12075.84	Gamma

**Table 2:** Cost-effectiveness of dabrafenib relative to dacarbazine and vemurafenib relative to dabrafenib.

	Total cost	Total effectiveness	Incremental cost	Incremental effectiveness	ICER
Dacarbazine	$15,221	0.1820 QALYs			
Dabrafenib	$38,547	0.3385 QALYs	$23,325	0.1565 QALYs	$149,042
Vemurafenib	$49,938	0.2905 QALYs	Dominated	Dominated	Dominated

QALYs: quality-adjusted life years; ICER: incremental cost-effectiveness ratio.

Note: comparisons are to the next least costly alternative.

**Table 3:** Base case cost-effectiveness of vemurafenib relative to dacarbazine.

	Total cost	Total effectiveness	Incremental cost	Incremental effectiveness	ICER
Dacarbazine	$15,221	0.1820 QALYs			
Vemurafenib	$49,938	0.2905 QALYs	$34,717	0.1085 QALYs	$319,972

QALYs: quality-adjusted life years; ICER: incremental cost-effectiveness ratio.

Note: comparison is to the chemotherapy drug option.
